# Supplementary material for: Analysis of the current status of knowledge, attitudes, and practices among stroke-related healthcare professionals in the treatment of shoulder pain in hemiplegic patients
Source: PeerJ. 2024 Dec 16;12:e18684. doi: 10.7717/peerj.18684 (PMC11657197; doi:10.7717/peerj.18684)
Supplement: Supplemental Information 2 [file peerj-12-18684-s002.docx]

**Stroke-Related Department Medical Staff Questionnaire Survey**

This questionnaire will conduct an anonymous survey of medical staff in stroke-related departments. Please read the questions carefully and answer truthfully

1. Basic Information:

- Your Gender:

- Your Department:

- Your Year of Employment:

- Your Professional Title:

- Your Position (Doctor, Nurse, etc.):

- How many years have you worked in your current department:

- Your Level of Education:

Knowledge Section

2. Do you know which situations can cause hemiplegic shoulder pain?

A. Fully Mastered B. Familiar C. Understand D. Partially Understand E. Completely Unaware

3. Do you know the incidence rate of hemiplegic shoulder pain?

A. Fully Mastered B. Familiar C. Understand D. Partially Understand E. Completely Unaware

4. Do you know about the proper positioning of the limb?

A. Fully Mastered B. Familiar C. Understand D. Partially Understand E. Completely Unaware

5. Do you know how to position the hemiplegic upper limb when sitting or standing?

A. Fully Mastered B. Familiar C. Understand D. Partially Understand E. Completely Unaware

6. Do you know how to determine if a patient has shoulder dislocation?

A. Fully Mastered B. Familiar C. Understand D. Partially Understand E. Completely Unaware

7. Do you know the definition of shoulder-hand syndrome?

A. Fully Mastered B. Familiar C. Understand D. Partially Understand E. Completely Unaware

8. Do you know how to alleviate hemiplegic shoulder pain?

A. Fully Mastered B. Familiar C. Understand D. Partially Understand E. Completely Unaware

9. Do you know the timing for wearing a shoulder splint?

A. Fully Mastered B. Familiar C. Understand D. Partially Understand E. Completely Unaware

10. Do you know the timing for using medication to relieve pain from a hemiplegic shoulder splint?

A. Fully Mastered B. Familiar C. Understand D. Partially Understand E. Completely Unaware

Attitude Section

11. Do you think rehabilitation education is meaningful?

A. Completely Willing B. Willing C. Indifferent D. Not Very Willing E. Completely Unwilling

12. Are you willing to educate patients on proper limb positioning?

A. Completely Willing B. Willing C. Indifferent D. Not Very Willing E. Completely Unwilling

13. Are you willing to instruct patients on wearing a shoulder splint?

A. Completely Willing B. Willing C. Indifferent D. Not Very Willing E. Completely Unwilling

14. Would you correct patients who are not wearing a shoulder splint correctly?

A. Completely Willing B. Willing C. Indifferent D. Not Very Willing E. Completely Unwilling

15. Do you think it is necessary to participate in learning about hemiplegic shoulder pain?

A. Completely Willing B. Willing C. Indifferent D. Not Very Willing E. Completely Unwilling

16. Are you willing to provide psychological counseling to patients with hemiplegic shoulder pain?

A. Completely Willing B. Willing C. Indifferent D. Not Very Willing E. Completely Unwilling

Behavior Section

17. Do you guide patients on proper limb positioning in your work?

A. Always B. Often C. Sometimes D. Occasionally E. Never

18. Do you instruct patients on wearing a shoulder splint in your work?

A. Always B. Often C. Sometimes D. Occasionally E. Never

19. Do you correct patients who are not wearing a shoulder splint correctly in your work?

A. Always B. Often C. Sometimes D. Occasionally E. Never

20. Do you assess the muscle strength of the hemiplegic side in your work?

A. Always B. Often C. Sometimes D. Occasionally E. Never

21. Do you assess the swelling condition of the hemiplegic limb in your work?

A. Always B. Often C. Sometimes D. Occasionally E. Never

22. Do you assess the pain condition of the hemiplegic limb in your work?

A. Always B. Often C. Sometimes D. Occasionally E. Never

23. Do you provide psychological counseling to patients with hemiplegic shoulder pain?

A. Always B. Often C. Sometimes D. Occasionally E. Never

24. Do you treat patients with hemiplegic shoulder pain?

A. Always B. Often C. Sometimes D. Occasionally E. Never
