# Supplementary material for: Analysis of the current status of knowledge, attitudes, and practices among stroke-related healthcare professionals in the treatment of shoulder pain in hemiplegic patients
Source: PeerJ. 2024 Dec 16;12:e18684. doi: 10.7717/peerj.18684 (PMC11657197; doi:10.7717/peerj.18684)
Supplement: Supplemental Information 3 [file peerj-12-18684-s003.docx]

**卒中相关科室医务人员问卷调查**

本问卷将对卒中相关科室医务人员进行不记名问卷调查，请各位老师仔细阅读题目后如实填写

1.基本信息：

您的性别：

您的科室：

您的入职年份:

您的职称：

您的岗位（医生、护理等）：

您在目前所在科室已工作几年：

您的文化程度：

知识部分

2.您知道哪些情况会导致偏瘫侧肩痛吗？

A.完全掌握 B.熟悉 C.了解 D.部分了解 E.完全不了解

3.您知道偏瘫肩痛的发生率吗?

A.完全掌握 B.熟悉 C.了解 D.部分了解 E.完全不了解

4.您知道良肢位摆放吗？

A.完全掌握 B.熟悉 C.了解 D.部分了解 E.完全不了解

5.您知道坐位或站立位时偏瘫上肢如何摆放吗？

A.完全掌握 B.熟悉 C.了解 D.部分了解 E.完全不了解

6.您知道如何判断患者是否有肩关节脱位吗？

A.完全掌握 B.熟悉 C.了解 D.部分了解 E.完全不了解

7.您知道肩手综合征的定义吗？

A.完全掌握 B.熟悉 C.了解 D.部分了解 E.完全不了解

8. 您知道如何缓解偏瘫肩痛吗？

A.完全掌握 B.熟悉 C.了解 D.部分了解 E.完全不了解

9. 您知道佩戴肩托的时机吗？

A.完全掌握 B.熟悉 C.了解 D.部分了解 E.完全不了解

10. 您知道偏瘫肩托应用药物止痛的时机吗

A.完全掌握 B.熟悉 C.了解 D.部分了解 E.完全不了解

态度部分

11. 您认为康复宣教是有意义的

A. 完全愿意 B. 愿意 C. 无所谓 D. 不太愿意 E. 完全不愿意

12. 您愿意对患者进行良肢位宣教

A. 完全愿意 B. 愿意 C. 无所谓 D. 不太愿意 E. 完全不愿意

13. 您愿意指导患者佩戴肩托

A. 完全愿意 B. 愿意 C. 无所谓 D. 不太愿意 E. 完全不愿意

14. 当您看到患者不正确佩戴肩托您愿意指正

A. 完全愿意 B. 愿意 C. 无所谓 D. 不太愿意 E. 完全不愿意

15. 您认为需要参加偏瘫肩痛相关知识的学习

A. 完全愿意 B. 愿意 C. 无所谓 D. 不太愿意 E. 完全不愿意

16. 您愿意对存在偏瘫肩痛的患者进行心理疏导吗

A. 完全愿意 B. 愿意 C. 无所谓 D. 不太愿意 E. 完全不愿意

行为部分

17. 您在工作中是否为患者指导良肢位摆放

A. 总是 B. 经常 C. 有时 D. 偶尔 E. 从不给予

18. 您在工作中是否为患者指导佩戴肩托

A. 总是 B. 经常 C. 有时 D. 偶尔 E. 从不给予

19. 您在工作中看到患者不正确佩戴肩托会指正吗

A. 总是 B. 经常 C. 有时 D. 偶尔 E. 从不给予

20. 您在工作中是否评估患者偏瘫侧肌力

A. 总是 B. 经常 C. 有时 D. 偶尔 E. 从不给予

21. 您在工作中是否评估患者偏瘫侧肢体肿胀情况

A. 总是 B. 经常 C. 有时 D. 偶尔 E. 从不给予

22. 您在工作中是否评估患者偏瘫侧肢体疼痛情况

A. 总是 B. 经常 C. 有时 D. 偶尔 E. 从不给予

23. 您会对存在偏瘫肩痛的患者进行心理疏导吗

A. 总是 B. 经常 C. 有时 D. 偶尔 E. 从不给予

24. 您会对存在偏瘫肩痛的患者进行治疗吗

A. 总是 B. 经常 C. 有时 D. 偶尔 E. 从不给予
